# The Level and Associated Factors of Creative Deviance of Clinical Nurses

**DOI:** 10.1002/nop2.70830

**Published:** 2026-09-14

**Authors:** Yan Rao, Dawei Jiang, Wenxian Xu

**Affiliations:** ^1^ School of Medicine QuZhou College of Technology Quzhou Zhejiang China; ^2^ Nursing Department Quzhou People's Hospital Quzhou Zhejiang China

**Keywords:** clinical nurses, creative deviance, nursing administration research, team psychological safety, workplace spirituality

## Abstract

**Aim:**

To examine the current level of creative deviance among clinical nurses and identify associated factors.

**Design:**

This study used a cross‐sectional design.

**Methods:**

A convenience sample of 712 clinical nurses was recruited. Data were collected using the Creative Deviance Scale, Workplace Spirituality Scale and Team Psychological Safety Climate Scale.

**Results:**

Overall, 650 valid questionnaires were returned, yielding a response rate of 91.3%. The mean creative deviance score among nurses was 17.78 (standard deviation = 2.86). Illness was negatively associated with creative deviance, whereas workplace spirituality and team psychological safety climate were positively associated with creative deviance.

**Conclusion:**

Creative deviance among clinical nurses was moderate. The findings suggest that health status, workplace spirituality and team psychological safety may be relevant to nurses' creative deviance. Longitudinal studies are needed to clarify causal relationships.

**Implications for the Profession:**

The findings provide preliminary evidence that may help nursing managers support creative deviance through targeted strategies. Managers may consider health monitoring programs, workplace spirituality initiatives, open dialogue, non‐punitive error reporting and formal channels that balance innovation with patient safety.

**Impact:**

- What Problem Did the Study Address?
  ○ Empirical research on creative deviance among clinical nurses remains limited.
- What Were the Main Findings?
  ○ Illness, workplace spirituality and team psychological safety climate were associated with creative deviance.
- Where and on Whom Will the Research Have an Impact?
  ○ The findings may inform nursing management and help nurse administrators foster innovation among clinical nurses.

**Reporting Method:**

This study adhered to the Strengthening the Reporting of Observational Studies in Epidemiology (STROBE) guideline for cross‐sectional studies.

**Patient or Public Contribution:**

No patient or public contribution was involved.

## 1 Background

Nurses are at the forefront of clinical practice and provide up to 80% of primary healthcare services. As the global disease landscape changes and healthcare technologies, care models, communication methods and data‐processing approaches continue to evolve, nurses frequently encounter unexpected situations that require innovative solutions (Abd‐Elmoghith et al. 2024). Nurses' innovative behaviours meet professional development needs and can improve nursing quality and patient safety (Barr et al. 2021).

However, standardized procedural management systems and resource shortages can limit the implementation of innovative behaviours. When ideas cannot be realized through formal channels, individuals may turn to informal channels. Innovative behaviour that violates reference norms without organizational knowledge but is intended to benefit the organization is called creative deviance, also known as bootlegging (Wang et al. 2023). Creative deviance is therefore a pro‐organizational behaviour characterized by deviation in behaviour but a reasonable purpose.

Previous research has shown that effective use of employees' deviant innovation can improve individual and team performance, enhance organizational innovation and support development over time (Kumar et al. 2024). Accordingly, creative deviance has received increasing research attention.

### 1.1 Theoretical Framework

Previous research has identified workplace spirituality and team psychological safety climate as potential factors influencing creative deviance (Zhu and Song 2023; Fu et al. 2022). To clarify whether these factors operate as environmental conditions or individual resources, this study draws on Social Cognitive Theory (Bandura 2001). According to this theory, behaviour results from dynamic interactions between personal factors and environmental events.

Within this framework, team psychological safety climate is conceptualized as a team‐level environmental factor that represents shared perceptions of interpersonal risk. Workplace spirituality is conceptualized as an individual‐level psychological resource that reflects a transcendent experience developed through meaningful work and connection with others. Creative deviance is conceptualized as behaviour that may arise from the interaction between this environmental factor and individual resource.

### 1.2 Workplace Spirituality as an Individual Resource

Creative deviance is challenging because individuals must address ongoing difficulties that require proactive initiative and resource support. Workplace spirituality refers to an individual's transcendent experience in the work context, nurtured through meaningful work and a sense of connection with others (Sode and Chenji 2024). According to Wang et al., workplace spirituality can be understood as a psychological dimension that extends beyond immediate work tasks. It can be fostered through meaningful work and a sense of belonging to a community, enabling individuals to experience deeper purpose and ultimate meaning in life (Wang et al. 2009). According to Conservation of Resources (COR) Theory, individuals strive to obtain, retain and protect valued resources (Hobfoll 1989). As an individual‐level psychological resource, workplace spirituality strengthens intrinsic motivation and may stimulate employee initiative and innovative ideas (Liu et al. 2024).

### 1.3 Team Psychological Safety as an Environmental Factor

The pressure and failure associated with creative deviance can lead to frustration, and personal self‐regulation alone may be insufficient for recovery. Social Cognitive Theory proposes interactions among environment, individuals and behaviour, with the environment guiding behaviour (Bandura 2001). Team psychological safety is defined as the shared perception among team members that they will not be penalized for speaking up, raising questions, asking for help, sharing ideas, making mistakes or expressing concerns (Marlow et al. 2025). Research has shown that team psychological safety, as a shared perception, is important for team performance, and sharedness among team members, termed team psychological safety climate strength, moderates this relationship (Fyhn et al. 2023). In nursing contexts, a study of 259 nurses in adult inpatient medical‐surgical units found that psychological safety was significantly correlated with safety climate perceptions, and both psychological safety and safety climate were related to nurses' self‐rated performance and perceptions of the care environment (Thurman Johnson et al. 2025). When psychological safety is high, individuals are less concerned about unfavourable reactions from their surroundings and can express their ideas and demonstrate different behaviours, thereby promoting boundary crossing and innovation. Conversely, when psychological safety is low, employees may worry about both the potential failure of creative deviance and the adverse consequences they may face, which may lead them to avoid such behaviours.

### 1.4 The Present Study

Research on creative deviance remains at an early stage, with limited reports in the nursing field. Two research gaps are particularly evident: (1) the current level of creative deviance among clinical nurses remains unknown; and (2) despite their potential relevance, factors associated with nurses' creative deviance, particularly workplace spirituality as an individual‐level resource and team psychological safety climate as an environmental factor, have not been systematically examined in nursing populations.

Given the cross‐sectional design and early stage of research on this topic, this study is hypothesis‐generating and aims to explore associations rather than infer causality. Therefore, this study evaluated the current level of creative deviance among clinical nurses and identified associated factors, with particular attention to workplace spirituality as an individual‐level psychological resource and team psychological safety climate as an environmental factor. The findings are intended to inform strategies that support nurses' creative deviance, foster innovative teams, and promote sustainable nursing development.

## 2 Methods/Methodology

### 2.1 Design

This study used an observational cross‐sectional design. This design was appropriate for exploring associations between variables without inferring causality. The methodology adhered to the Strengthening the Reporting of Observational Studies in Epidemiology (STROBE) Statement guidelines for cross‐sectional studies (Ribeiro et al. 2024). From May to June 2024, nurses from three tertiary general hospitals in Quzhou City were selected as survey participants. The hospitals represented different healthcare service models relevant to clinical creative deviance. Hospital A was a municipal comprehensive teaching hospital serving as the regional medical center with strict hierarchical management protocols. Hospital B was a specialized hospital integrating traditional and Western medicine, requiring adaptive care innovations across therapeutic paradigms. Hospital C was a psychiatric specialty hospital operating in a high‐risk environment where protocol deviations may address emergent patient safety issues. Convenience sampling was used to recruit participants who met the following criteria: (1) held a nurse qualification certificate and were registered; and (2) had worked for at least 1 year. Nurses engaged in further study, interns, and those unable to participate because of personal leave, sick leave, or other reasons were excluded.

The sample size was calculated using G*Power software (Faul et al. 2009) for multiple linear regression. Based on Cohen's criteria (Cohen 1988), a medium effect size (*f* ^2^ = 0.15) was assumed. With a significance level of α = 0.05, desired power of 0.80, and 16 predictors, the minimum required sample size was 143 participants. Anticipating a 10% invalid response rate, we aimed to recruit at least 158 participants. The final sample of 650 valid respondents exceeded this requirement and provided sufficient statistical power.

The completed STROBE checklist for cross‐sectional studies is available as Appendix S1.

### 2.2 Instruments With Validity and Reliability

#### 2.2.1 General Information Questionnaire

The general information questionnaire was developed after consultation with relevant literature and subject‐area experts. It collected information on sex, age, educational background, department, professional title, work experience, job category, marital status, annual income, monthly night‐shift frequency, weekly working hours, workplace violence, illness and work pressure.

The coding scheme for each variable was as follows:

- Gender: female = 1, male = 2
- Age: 20–30 years = 1, 31–40 years = 2, ≥ 41 years = 3
- Educational background: junior college or below = 1, bachelor degree or above = 2
- Work department: medical = 1, surgery = 2, emergency and ICU = 3, other = 4
- Professional title: senior nurse = 1, supervisor nurse = 2, co‐chief nurse or above = 3
- Working experience: ≤ 10 years = 1, 11–20 years = 2, 21–30 years = 3, ≥ 31 years = 4
- Job category: permanently employed = 1, temporarily employed = 2
- Marital status: unmarried = 1, married = 2
- Annual income (yuan): < 150,000 = 1, 150,000–200,000 = 2, > 200,000 = 3
- Monthly night shift frequency: 0 shifts = 1, ≤ 8 shifts = 2, ≥ 9 shifts = 3
- Weekly working hours: < 40 h = 1, 40–44 h = 2, ≥ 45 h = 3
- Workplace violence: no = 0, yes = 1
- Illness: no = 0, yes = 1

Work pressure was assessed using a single‐item self‐rating scale ranging from 0 to 10, where 0 indicated no stress and 10 indicated maximum stress. Single‐item stress and burnout measures have been validated in healthcare worker populations. A systematic review reported sufficient criterion validity for single‐item burnout measures, with an area under the curve of 0.75–0.92 and sensitivity of 71%–84% (Emal et al. 2023). According to criteria proposed by CaoJing (Cao 2022), scores of 0–3 indicated low pressure, scores of 4–6 indicated moderate pressure, and scores of 7–10 indicated high pressure. These categories were coded as low = 1, moderate = 2, and high = 3.

#### 2.2.2 Creative Deviance Scale

The Creative Deviance Scale was developed by Criscuolo et al. (Criscuolo et al. 2014) and includes five items. The Chinese version underwent cross‐cultural adaptation, including forward translation, back‐translation, reconciliation, and expert review, with a content validity index of 0.91. Each item is rated on a 5‐point scale from 1, strongly disagree, to 5, strongly agree. Total scores range from 5 to 25, with higher scores indicating greater creative deviance among nurses. The reliability and validity of this scale have been tested in relevant Chinese literature (Shi 2023). In this study, Cronbach's α coefficient was 0.897. To date, no established norms for this scale have been published for the Chinese nursing population. Therefore, the theoretical midpoint was used as a reference for score interpretation. The mean per‐item score in this sample was 3.55, which was above the theoretical midpoint and indicated a moderate level.

#### 2.2.3 Workplace Spirituality Scale

The Workplace Spirituality Scale was developed in Chinese by KeJianglin et al. (Ke et al. 2014) and consists of 27 items across three dimensions: meaningful work, sense of community, and alignment between organizational and individual values. Each item is rated on a 5‐point scale from 1, strongly disagree, to 5, strongly agree. Total scores range from 27 to 135, with higher scores indicating higher workplace spirituality among nurses. The original validation confirmed structural validity, with confirmatory factor analysis results of *χ* ^2^/df = 1.29, Comparative Fit Index = 0.93, and Root Mean Square Error of Approximation = 0.052. In this study, Cronbach's α coefficient was 0.983.

#### 2.2.4 Team Psychological Safety Climate Scale

The Chinese version of the Team Psychological Safety Climate Scale was developed by WuZhiping (Wu and Chen 2011) and consists of 16 items across four dimensions: direct expression of opinions, mutual respect, interpersonal risk‐taking, and mutual trust. Each item is rated on a 5‐point scale from 1, strongly disagree, to 5, strongly agree. Total scores range from 16 to 80, with higher scores indicating a more favourable team psychological safety climate as perceived by nurses. The original validation confirmed structural validity, with confirmatory factor analysis results of *χ* ^2^/df = 2.17, Comparative Fit Index = 0.915, and Root Mean Square Error of Approximation = 0.007. In this study, Cronbach's α coefficient was 0.911.

### 2.3 Data Collection and Quality Control

Before data collection, eligible nurses received information about the study purpose, significance, and questionnaire‐completion procedures. Written informed consent was obtained from nurses who agreed to participate. Only after consent had been obtained was the questionnaire QR code provided. Participants were instructed to complete the questionnaire anonymously and independently. To prevent duplicate submissions, each Internet Protocol address was restricted to one response. To ensure questionnaire completeness, all items were set as required fields.

After questionnaire collection, the following quality‐control procedures were applied. Questionnaires with contradictory or logically inconsistent responses were excluded. Questionnaires in which more than 50% of responses were identical were also excluded as likely invalid. Given the questionnaire length of 62 items and an average adult reading speed of approximately 200–250 words per minute, a minimum completion time of 3 min was estimated as necessary for participants to read each item, consider the appropriate response, and make a selection. Therefore, questionnaires completed in less than 3 min were excluded because of the high likelihood of invalid responses. This threshold was not predetermined through a pilot study, which is acknowledged as a limitation.

### 2.4 Data Analysis

Statistical analyses were conducted using SPSS version 27.0. Categorical variables were summarized as frequencies and percentages. Normality was assessed using the Shapiro–Wilk and Kolmogorov–Smirnov tests and confirmed visually using Q‐Q plots. Normally distributed continuous variables were presented as mean (standard deviation) and analysed using parametric tests, including the independent‐samples *t*‐test for two groups and one‐way analysis of variance for three or more groups. Non‐normally distributed variables were presented as median (interquartile range) and analysed using non‐parametric tests, including the Mann–Whitney *U* test for two groups and the Kruskal–Wallis H test for three or more groups. Pearson or Spearman correlation analyses were conducted as preliminary descriptive analyses to examine bivariate associations among the main study variables. Multivariable linear regression was used to identify factors independently associated with creative deviance after adjustment for potential confounders. Regression assumptions were assessed using residual scatterplots for linearity, the Durbin‐Watson test for independence of errors, the Shapiro–Wilk test, Q‐Q plots, and histograms for normality of residuals, the Breusch‐Pagan test and residual plots for homoscedasticity, and variance inflation factors for multicollinearity. Harman's single‐factor test was performed using all items from the three scales. The first unrotated factor explained 48.476% of the total variance, which was below the 50% threshold and indicated no serious common method bias. A two‐tailed *p* value < 0.05 was considered statistically significant. Given the hypothesis‐generating nature of this study, no adjustment for multiple testing was applied, and the risk of false‐positive findings is acknowledged as a limitation.

### 2.5 Ethical Considerations

This study was approved by the Ethics Committee of the authors' affiliated university (Approval No. 2024081811) and was conducted in accordance with the Declaration of Helsinki. Recruitment was conducted using neutral flyers distributed by unit managers to eligible nurses. Independent researchers then screened nurses for eligibility and excluded those on leave. The informed consent process was completed in two stages. In Stage 1, before any data collection, all eligible nurses were invited to a face‐to‐face meeting. Independent researchers provided a verbal explanation of the study purpose, procedures, risks, and benefits. Nurses were given 48 h to consider whether to participate. Those who agreed signed a written informed consent form.

In Stage 2, after written consent had been obtained, participants received a unique QR code that directed them to the online questionnaire platform. The QR code was used only to provide convenient access to the questionnaire and did not replace or bypass the written consent process. No data were collected before written consent was obtained.

Participants had the unconditional right to withdraw at any time without giving a reason, and six nurses exercised this right. Post‐study debriefing reports were available on request. The following measures were implemented to protect participant privacy. First, raw data were de‐identified immediately. Second, coded storage was used instead of personal identifiers. Third, hospital names were anonymized as Site A, Site B, and Site C. Fourth, data will be destroyed after a 3‐year retention period in accordance with institutional policy.

## 3 Results

### 3.1 Subjects

A total of 712 questionnaires were distributed. After verification by two independent researchers, 650 valid questionnaires were retained, yielding an effective response rate of 91.3%.

Participants were predominantly female (96.5%), with a mean age of 32.13 (standard deviation = 7.64) years (range: 20–59) and mean work experience of 10.73 (standard deviation = 7.98) years (range: 1–40); Most nurses held bachelor's degrees or higher (82.0%); Senior nurses constituted the largest professional group (55.5%). 65.1% were permanently employed; Nearly half (49.7%) reported ≤ 8 night shifts/month 37.8% worked 40–44 h/week, while 25.8% exceeded 45 h; 27.8% experienced workplace violence; Medical departments represented the largest work setting (42.9%); High work pressure was reported by 37.2%; Detailed characteristics are summarized in Table 1, including income distribution (70.2% earned < ¥150,000 annually), marital status (58.9% married), and health status (14.2% reported illnesses).

**TABLE 1:** The general data of subjects and results of univariate analysis of Creative Deviance (*n* = 650).

Demographics	*n* (%)	Creative deviance (M (SD)/Median (IQR))	*t*/*F*/*Z*/*H*	*p*
Gender^c^				
Female	627 (96.5)	17.76 (2.85)	−1.867	0.062
Male	23 (3.5)	18 (18–21)		
Age (years)^d^				
20–30	336 (51.7)	18 (16–20)	0.585	0.746
31–40	218 (33.5)	17.80 (3.03)		
≥ 41	96 (14.8)	17.19 (3.27)		
Educational Background^a^				
Junior college or below	117 (18.0)	17.48 (3.02)	−1.278	0.202
Bachelor degree or above	533 (82.0)	17.85 (2.82)		
Work department^d^				
Medical	279 (42.9)	18 (16–19)	5.998	0.112
Surgery	209 (32.2)	18 (16–21)		
Emergency and ICU	100 (15.4)	18 (15–20)		
Other	62 (9.5)	17.00 (3.52)		
Professional title^b^				
Senior nurse	361 (55.5)	17.71 (2.81)	1.556	0.212
Supervisor nurses	210 (32.3)	17.70 (3.10)		
Cochief nurses or above	79 (12.2)	18.32 (2.36)		
Working experience (years)^b^				
≤ 10	379 (58.3)	17.69 (2.83)	0.433	0.730
11–20	196 (30.2)	17.86 (3.04)		
21–30	56 (8.6)	18.11 (2.49)		
≥ 31	19 (2.9)	17.95 (2.61)		
Job category^a^				
Permanently employed	423 (65.1)	17.79 (2.92)	0.089	0.929
Temporarily employed	227 (34.9)	17.77 (2.76)		
Marital status^a^				
Unmarried	267 (41.1)	17.54 (2.80)	−1.828	0.068
Married	383 (58.9)	17.96 (2.89)		
Annual income^b^				
< 150,000	456 (70.2)	17.59 (2.93)	3.663	0.026^e^
150,000–20,000	168 (25.8)	18.23 (2.74)		
> 200,000	26 (4.0)	18.35 (2.08)		
Monthly night shift frequency^b^				
0	154 (23.7)	17.41 (2.85)	5.538	0.004^f^
≤ 8	323 (49.7)	18.16 (2.73)		
≥ 9	173 (26.6)	17.42 (3.03)		
Weekly working hours^b^				
< 40	236 (36.3)	18.27 (2.64)	6.891	0.001^g^
40–44	246 (37.8)	17.71 (2.81)		
≥ 45	168 (25.8)	17.21 (3.13)		
Workplace violence^a^				
None	469 (72.2)	18.02 (2.77)	3.391	0.001
Yes	181 (27.8)	17.18 (3.02)		
Illness^a^				
None	558 (85.8)	17.94 (2.84)	3.376	0.001
Yes	92 (14.2)	16.86 (2.82)		
Work pressure^b^				
Low level	119 (18.3)	17.69 (2.93)	3.663	0.026^h^
Medium level	289 (44.5)	18.23 (2.74)		
High level	242 (37.2)	18.35 (2.08)		

^a^ Independent sample *t*‐test.

^b^ One‐way ANOVA test.

^c^ Mann–Whitney *U* test.

^d^ Kruskal–Wallis H Test.

^e^ Post hoc test: < 15 versus 15 ~ (*p* = 0.012).

^f^ Post hoc test: 0 versus ≤ 8 (*p* = 0.007), ≤ 8 versus ≥ 9 (*p* = 0.006).

^g^ Post hoc test: < 40 versus 40 ~ (*p* = 0.032), < 40 versus ≥ 45 (*p* < 0.001).

^h^ Post hoc test: low level versus high level (*p* < 0.001); medium level versus high level (*p* < 0.001).

### 3.2 Univariate Analysis of Nurses' Creative Deviance

Univariate analysis showed that creative deviance scores differed significantly across six variables: illness, monthly night‐shift frequency, weekly working hours, annual salary, workplace violence, and work pressure (Table 1). Nurses reporting illness showed lower creative deviance scores 16.86 (standard deviation = 2.82) vs. no illness 17.94 (standard deviation = 2.84), *t* = 3.376, *p* = 0.001; One‐way ANOVA test indicated significant differences across groups (*F* = 5.538, *p* = 0.001): 0 shifts/month17.41 (standard deviation = 2.85) ≤ 8 shifts/month 18.16 (standard deviation = 2.73), ≥ 9 shifts/month 17.42 (standard deviation = 3.03); Scores decreased with longer Weekly Working hours (*F* = 6.891, *p* = 0.001): < 40 h: 18.27 (standard deviation = 2.64), 40–44 h:17.71 (standard deviation = 2.81), ≥ 45 h: 17.21 (standard deviation = 3.13); Higher income associated with higher scores (*F* = 3.663, *p* = 0.026); Experienced violence 17.18 ± 3.02, No violence 18.02 (standard deviation = 2.77), *t* = 3.391, *p* = 0.001; Scores increased with higher Work Stress levels (*F* = 3.663, *p* = 0.026): Low level 17.69 (standard deviation = 2.93), Medium level 18.23 (standard deviation = 2.74), High level 18.35 (standard deviation = 2.08).

### 3.3 Correlations Among Nurses' Creative Deviance, Workplace Spirituality and Team Psychological Safety Climate

As a preliminary analysis, Pearson or Spearman correlation coefficients were calculated to examine bivariate associations among the main study variables. The correlation matrix is shown in Table 2. Nurses' creative deviance was positively correlated with workplace spirituality (*r* = 0.556, *p* < 0.001) and team psychological safety climate (*r* = 0.524, *p* < 0.001). Scores of Nurses' Creative Deviance Scale, Workplace Spirituality Scale, and Team Psychological Safety Climate Scale are shown in Table 3.

**TABLE 2:** Correlation matrix among creative deviance, workplace spirituality and team psychological safety climate.

Variable	1	2	3
1. Creative deviance	—		
2. Workplace spirituality	0.556**	—	
3. Team psychological safety climate	0.524**	0.644**	—

*Note:* Pearson correlation was used for normally distributed variables; Spearman correlation was used for non‐normally distributed variables.

** *p* < 0.001.

**TABLE 3:** Creative Deviance, Workplace Spirituality and Team Psychological Safety Climate of nurses (*n* = 650).

Variables	Items	Total score	Per item score
		Mean (SD)	Mean (SD)
Creative Deviance Scale	5	17.78 (2.86)	3.55 (0.57)
Workplace Spirituality Scale	27	105.62 (20.25)	3.91 (0.75)
Meaningful work	9	33.09 (7.72)	3.68 (0.86)
Sense of community	9	36.02 (6.72)	4.0 (0.75)
Alignment between the organization's and individual's values	9	36.51 (7.17)	4.06 (0.80)
Team Psychological Safety Climate Scale	16	58.52 (6.28)	3.66 (0.39)
Direct expression of opinions	4	14.27 (1.90)	3.57 (0.47)
Mutual respect	4	16.52 (2.72)	4.13 (0.68)
Interpersonal risk‐taking	4	12.09 (2.83)	3.02 (0.71)
Mutual trust	4	15.64 (2.56)	3.91 (0.64)

### 3.4 Multivariable Linear Regression Analysis of Nurses' Creative Deviance

Multivariable linear regression was performed to identify factors independently associated with creative deviance among clinical nurses. The independent variables were illness, monthly night‐shift frequency, weekly working hours, annual salary, workplace violence, work pressure, workplace spirituality and team psychological safety climate.

Categorical variables were coded as follows:

- Illness: None (reference = 0), Yes = 1.
- Workplace violence: None (reference = 0), Yes = 1.
- Work pressure: low level = 1, medium level = 2, high level = 3 (treated as an ordinal variable).
- Monthly night shift frequency: 0 shifts = 1, ≤ 8 shifts = 2, ≥ 9 shifts = 3.
- Weekly working hours: < 40 h = 1, 40 ~ hours = 2, ≥ 45 h = 3.
- Annual salary: < 150,000 yuan = 1, 150,000 ~ yuan = 2, ≥ 200,000 yuan = 3.

Workplace spirituality and team psychological safety climate scale scores were entered as continuous variables with their original values.

All regression assumptions were adequately met (or no serious violations were detected). Scatterplots confirmed linear relationships. The Durbin‐Watson statistic was 1.920, indicating independence of residuals. Because of the large sample size (*n* = 650), the Shapiro–Wilk test was significant (*p* < 0.01). However, visual inspection of the histogram, which was approximately symmetric, and the Q‐Q plot, in which points followed the diagonal, supported approximate normality. Similarly, the Breusch‐Pagan test was significant (*p* < 0.01), likely because of the large sample size, but visual inspection of the residual plots showed no clear heteroscedastic pattern. All variance inflation factors were below 2, ranging from 1.034 to 1.785, indicating no multicollinearity.

The regression results showed that illness was negatively associated with creative deviance, whereas workplace spirituality and team psychological safety climate were positively associated with creative deviance (all *p* < 0.01). Other variables, including work pressure, workplace violence, monthly night‐shift frequency, weekly working hours, and annual salary, were not significantly associated with creative deviance in the regression model (*p* > 0.05) (Table 4).

**TABLE 4:** Multiple linear regression analysis of factors associated with creative deviance among nurses (*n* = 650).

Independent variables	*b*	SE	*β*	*t*	*p*	95% CI	
						Lower CI	Upper CI
Constant	5.570	1.130	—	4.931	< 0.001	3.352	7.789
Workplace Spirituality	0.051	0.006	0.364	8.665	< 0.001	0.040	0.063
Team Psychological Safety Climate	0.123	0.019	0.271	6.537	< 0.001	0.086	0.160
Illness (ref = None)							
Yes	−0.570	0.264	−0.070	−2.159	0.031	−1.089	−0.051
Annual income	0.174	0.166	0.034	1.049	0.295	−0.152	0.499
Monthly night shift frequency	0.246	0.131	0.061	1.886	0.060	−0.010	0.503
Weekly working hours	−0.162	0.124	−0.044	−1.304	0.193	−0.406	0.082
Workplace violence (ref = None)							
Yes	−0.083	0.214	−0.013	−0.386	0.699	−0.502	0.337
Work pressure	−0.095	0.138	−0.024	−0.687	0.492	−0.367	0.177

*Note:* *R* ^2^ = 0.368, Adjusted *R* ^2^ = 0.360, *F* = 41.485, *p* < 0.001, VIF = 1.034–1.785, Durbin‐Watson = 1.920. Dummy variables: Illness (reference: None, coded as 0); Workplace violence (reference: None, coded as 0). Other categorical variables (work pressure, monthly night shift frequency, weekly working hours, annual salary) were entered as ordinal variables. Continuous variables (workplace spirituality and team psychological safety climate) were entered with their original scores.

## 4 Discussion

### 4.1 Nurses' Creative Deviance Is at a Medium Level

The mean Creative Deviance Scale score was 17.78 (standard deviation = 2.86), and the mean per‐item score was 3.55 (standard deviation = 0.57). This score was above the theoretical midpoint of 3.00, indicating a moderate level of creative deviance. However, it was lower than the score reported among employees in education, finance, and public utilities, whose mean score was 4.10 (standard deviation = 0.97) (Zhu and Song 2023). This finding is consistent with recent studies reporting moderate levels of innovative behaviour among clinical nurses across different samples and countries (Liu et al. 2025; Li et al. 2025). Several nursing‐specific factors may explain why nurses' creative deviance scores were lower than those reported in other professions. First, the hierarchical structure of nursing, with clear chains of command and direct supervision, may discourage bottom‐up innovation, whereas professionals in other fields may experience flatter organizational structures (Dillard‐Wright 2026). Second, nurses face systemic barriers in the innovation process, including unfamiliar innovation ecosystems, lengthy development processes that may lead to burnout, fear of idea theft, and difficulties with commercialization (van der Zanden et al. 2025). Third, organizational support for nurse‐led innovation remains insufficient, with nursing schools receiving disproportionately low research funding and nurse‐scientists often lacking protected time and resources (Dzikowicz et al. 2024).

Unlike planned innovative behaviour, creative deviance refers to independent innovative behaviour undertaken by individuals who believe that their actions can benefit the organization. It may therefore provide an important pathway through which organizations achieve unexpected innovation (Lyu et al. 2022). Clinical nurses commonly report concerns about insufficient hospital support and investment in nurses' innovative behaviours (Olivé et al. 2026). Therefore, nursing managers should adopt a balanced approach to the tension between creative deviance and formal innovation pathways. Managers should communicate proactively with nurses to understand their perspectives on innovation and provide incremental guidance, instrumental support, and emotional support.

### 4.2 Nurses Who Did Not Suffer From Illness Had a Higher Level of Creative Deviance

The results showed that nurses without illness had higher levels of creative deviance (*p* = 0.017). Better health may enhance nurses' capacity to cope with stress and complete tasks efficiently, and it may also contribute to greater work engagement (Chen, Kewou, et al. 2024). Previous research has shown that individuals with greater work engagement have more energy and motivation at work and may be more likely to engage in creative deviance to protect organizational interests (Petersen et al. 2021). Therefore, nursing managers should advocate health‐promoting behaviours, such as regular exercise and balanced diets, and hospitals should consider providing supportive facilities. In addition, assigning work tasks scientifically to reduce unnecessary physical strain may help maintain nurses' health and encourage creative deviance.

### 4.3 Higher Workplace Spirituality Is Associated With Higher Creative Deviance Among Nurses

Workplace spirituality is characterized by the self‐transcendence that individuals experience when they recognize the significance of their work and the values upheld by their group and organization. This advanced spiritual resource in the workplace helps individuals cope effectively with daily tasks as well as challenging and risky work (Moez et al. 2024). In this study, workplace spirituality was positively associated with nurses' creative deviance, with a positive correlation between the two variables (*r* = 0.556, *p* < 0.001). This finding indicates that stronger workplace spirituality is associated with higher creative deviance among nurses and is consistent with the findings of Yongyue et al. (Zhu and Song 2023). Although creative deviance is intended to enhance organizational efficiency, it lacks formal organizational authorization. Individuals must therefore bear uncertainty about their investment and the high risk of unfavourable outcomes (Lyu et al. 2022). Compared with general innovative behaviour, nurses' creative deviance may require stronger internal motivation and commitment. According to Conservation of Resources Theory (Chen, Wang, et al. 2024), nurses with high workplace spirituality may obtain more spiritual resources, such as job satisfaction, organizational identity, and attachment, from their work, groups, and organizations. These accumulated resources may motivate them to engage in uncertain innovative activities, including creative deviance, to support organizational development. Consequently, nursing managers should recognize the importance of workplace spirituality and may consider evaluating it when recruiting for roles that require innovation. Training and initiatives designed to enhance spiritual resources at the work, group, and organizational levels may also encourage nurses' creative deviance.

### 4.4 Better Team Psychological Safety Climate Is Associated With Higher Creative Deviance Among Nurses

Perceived psychological safety is a fundamental condition for encouraging innovative behaviour. A team with a strong psychological safety climate enables members to perceive interpersonal risk‐taking as acceptable, creating a supportive and tolerant atmosphere that can foster individual innovative behaviour (Yin et al. 2022). In this study, team psychological safety climate was positively associated with nurses' creative deviance, with a positive correlation between the two variables (*r* = 0.524, *p* < 0.001). This finding indicates that a higher level of team psychological safety climate co‐occurs with higher creative deviance among nurses. Maslow's hierarchy of needs theory has been applied to understand nurses' workplace needs, with psychological safety and belonging identified as foundational requirements before higher‐level needs, such as esteem and self‐actualization, can be addressed (Raso 2022). On one hand, creative deviance involves many unknown risks and obstacles. A safe and harmonious team that fosters mutual assistance and tolerance can improve individuals' capacity to manage creative deviance. In such a climate, employees may perceive that they will not be punished even if they engage in unauthorized innovative behaviours and encounter setbacks, which may encourage them to pursue innovative practices (Peng and Chen 2023). On the other hand, psychological safety in the workplace enables individuals to concentrate more fully and feel more secure in their work, which may strengthen organizational identification and motivation to support organizational development (Berraies and Chouiref 2023). Therefore, nursing managers should foster sincere cooperation, open communication, and non‐punitive error handling within teams to create a safe and harmonious atmosphere that supports innovative practice among nurses.

### 4.5 Balancing Innovation and Patient Safety

Nursing is a high‐risk field in which operating outside established norms may compromise patient safety. Therefore, this study does not advocate individual rule‐breaking. Rather than encouraging individual risk‐taking, organizations should foster a psychologically safe climate that enables nurses to voice concerns through official channels (Seo and Lee 2024). This approach aligns innovation with patient safety, the core mission of nursing. The positive deviance approach, which has been shown to reduce healthcare‐associated infections (Kassie et al. 2024; Clark et al. 2024), also supports fostering supportive climates rather than encouraging individual violations.

Collectively, these evidence‐informed recommendations, including health monitoring, workplace spirituality initiatives, team psychological safety, and balancing innovation with patient safety, may be transferable across diverse nursing contexts and relevant to nurse managers, educators, and policymakers internationally.

### 4.6 Limitations

This study has several limitations. First, the cross‐sectional design precludes causal inference; therefore, all identified associations should be interpreted as correlational. Future longitudinal studies are needed to examine causal relationships.

Second, the study was conducted within one provincial healthcare system in China and used convenience sampling, which limits generalizability. Inter‐hospital differences were not compared, and the extent to which creative deviance varies across different hospital types remains unknown. Future multicenter studies should include diverse geographical regions and hospital settings. Third, several measurement‐related limitations should be considered. First, work pressure was assessed using a single‐item scale; multi‐item scales would provide a more comprehensive assessment. Second, the 3‐min exclusion threshold for questionnaires was based on estimation rather than a pilot study. We did not perform a sensitivity analysis comparing results with and without excluded fast responders, defined as those who completed the questionnaire in < 3 min, because this cutoff was not established through a pilot study. Third, no established normative data for the Creative Deviance Scale are available for the Chinese nursing population; therefore, the interpretation of a moderate level was based on the theoretical midpoint of 3.0. Fourth, although Harman's single‐factor test indicated no serious common method bias, with the first factor explaining 48.476% of the variance, self‐reported data may still introduce bias. Fourth, multiple statistical tests were conducted without adjustment for multiple comparisons. Given the hypothesis‐generating nature of this study, the findings should be interpreted as exploratory.

## 5 Conclusion

These findings may have relevance for nursing management internationally. Given the observed moderate level of creative deviance among clinical nurses, nurse leaders may consider the following evidence‐informed approaches.

Proactive health monitoring may be useful because nurses in better health reported higher creative deviance. Managers could support health‐promoting behaviours and work schedules that reduce unnecessary strain.

Cultivating workplace spirituality may also be beneficial. Given its positive association with creative deviance, fostering values alignment and purpose‐driven practices, adapted to local cultural contexts, may encourage innovation.

Fostering psychological safety may help support creative deviance. Because team psychological safety climate was associated with creative deviance, promoting open dialogue and non‐punitive error reporting represents a promising managerial approach.

Balancing innovation and patient safety remains essential. Rather than encouraging individual nurses to operate outside organisational norms, nursing managers should foster a psychologically safe climate in which nurses feel able to voice concerns and propose innovative solutions through official channels. This approach aligns innovation with patient safety, the core mission of nursing.

These strategies should be interpreted as evidence‐informed possibilities derived from this hypothesis‐generating study rather than as definitive causal conclusions. International collaborations should develop standardized frameworks to further test and implement these approaches.

## Author Contributions

**Yan Rao:** conceptualization, methodology, writing – original draft, funding acquisition, data curation, project administration. **Dawei Jiang:** methodology, data curation, investigation, software, validation, writing – review and editing. **Wenxian Xu:** writing – review and editing, conceptualization, validation.

## Funding

This research was supported by the First Batch of Teaching Reform Projects for Vocational Education in Zhejiang Province during the 14th Five‐Year Plan Period (Grant No. 2026SCG333).

## Disclosure

Patient and Public Involvement: It was not appropriate or possible to involve patients or the public in the design, conduct, reporting, or dissemination plans of this research.

Statistical Declaration: The authors confirm that this submission conforms to the Journal's statistical guidelines.

The statistical analyses were conducted and reviewed by the authors; co‐author Dawei Jiang has statistical expertise. No external statistician was consulted.

The authors affirm that the statistical methods applied are appropriate for the data and study design, and the results have been interpreted correctly.

AI Use Statement: No artificial intelligence (AI) tools were used in the design, data analysis, or interpretation of this study. AI tools were used solely for English language polishing during manuscript preparation.

## Ethics Statement

This study was approved by the Ethics Committee of QuZhou College of Technology (Approval No. 2024081811). All methods were performed in accordance with the Declaration of Helsinki.

## Consent

All participants provided written informed consent and volunteered to participate.

## Conflicts of Interest

The authors declare no conflicts of interest.

## Supporting information


**Appendix S1:** STROBE Statement—Checklist of items that should be included in reports of cross‐sectional studies.

## Data Availability

The data that support the findings of this study are available from the corresponding author upon reasonable request.
